# The World Health Organization and global smallpox eradication

**DOI:** 10.1136/jech.2006.055590

**Published:** 2008-09-08

**Authors:** S Bhattacharya

## Abstract

**Background:**

This article examines the multifaceted structures and complex operations of the World Health Organization and its regional offices; it also reassesses the form and the workings of the global smallpox eradication programme with which these bodies were closely linked in the 1960s and 1970s.

**Methods:**

Using the case study of South Asia, it seeks to highlight the importance of writing nuanced histories of international health campaigns through an assessment of differences between official rhetoric and practice.

**Results and conclusion:**

The article argues that the detailed examination of the implementation of policy in a variety of localities, within and across national borders, allows us to recognise the importance of the agency of field managers and workers. This analytical approach also helps us acknowledge that communities were able to influence the shape and the timing of completion of public health campaigns in myriad ways. This, in turn, can provide useful pointers for the design and management of health programmes in the contemporary world.

The global eradication of smallpox was certified by an independent committee of experts in December 1979, and the announcement was ratified by the World Health Organization (WHO) in 1980. Widely hailed as one of the biggest medical triumphs of the twentieth century, the campaign to eradicate smallpox worldwide is often described in overly simplistic terms in institutional histories, published memoirs and, not least, academic works that unquestioningly accept participants’ retrospective retelling of their experiences. The picture presented, generally, is one of unified actions; the many different cogs of a complex administrative wheel, it is frequently claimed, apparently worked in almost perfect unison, causing orders from the top of the organisational pyramid to be implemented in localities across the globe. Such an interpretation would have us believe that the calculations and actions of a few senior managers controlled the actions of a huge number of public health and medical personnel of different educational backgrounds, nationalities, political affiliations and gender, over the course of more than a decade. This perspective downplays the ability of field workers to come up with ideas and implement them according to the plethora of social, political and economic conditions encountered in a multiplicity of localities.^1^^–^3

The organised drive to expunge smallpox was, however, a much more complicated and disjointed entity. A composite of numerous multifaceted country- and region-oriented public health programmes, the campaign combined the work of several non-government agencies with that of different national, provincial and district administrations. A careful assessment of unpublished WHO papers reveals that these collaborative ventures involved a series of time-consuming negotiations with numerous bureaucrats, politicians and funding agencies. This, in turn, resulted in complex administrative and financial arrangements that needed to be re-established at frequent intervals; a product of the fact that inter- and intra-governmental discussions and the resulting aid packages, which were to prove decisive to the successful completion of smallpox eradication, were frequently organised on varying bilateral and multilateral bases. WHO officials were generally involved in multilateral negotiations, as initiators of negotiations, witnesses to the completion of signed agreements and, sometimes, apolitical distributors of resources in the shape of vaccine, vaccinating kits, money and personnel.^4^

Yet, these WHO representatives were often not in control of the unfolding of policy imperatives, mainly because a variety of international, regional, national and local developments continually threatened to blow the most tightly organised plans off course. Projects often stuttered along uncharted paths, as their managers were constantly forced to adapt to unexpected problems. Because of this, desired results were frequently achieved almost accidentally, surprising even the most optimistic and committed field personnel. An appreciation of all these complexities, which are very often glossed over, does not detract from the significance of the smallpox eradication. To the contrary, it highlights the enormity of the achievement, which many officials and politicians considered impossible during the 1960s and 1970s.^4^ ^5^

## THE ADMINISTRATIVE COMPLEXITY OF THE WHO

The United Nations came into being soon after the end of the Second World War, and the WHO was established as one of its major, specialised sections in 1948. The WHO headquarters (HQ) was established in Geneva, Switzerland, and this body took on the role of trying to help in the development and coordination of public health and medical schemes across the globe. In its formative years, these activities were targeted particularly at regions that had been badly affected by the war and countries that had managed to break loose from colonialism; the advertised goal was to carry out all this work on an apolitical basis.^6^ ^7^

The WHO has, from its inception, been a complex administrative structure. It consists of a Health Assembly, a Director General’s office that is in regular touch with a relatively tightly knit advisory committee and, not least, a large secretariat. The Assembly was formed by representatives of all the member nations, who met at regular intervals in Geneva and involved themselves in proposing schemes and voting for their implementation. This body was given the power, through the WHO constitution, to ask the Director General’s office and his/her advisory committee to develop detailed plans for the implementation of policies and programmes; all completed plans were presented to the assembly and then forwarded to the secretariat’s bureaucracy for implementation. This, in turn, ensured the formulation of numerous plans within the WHO HQ and the various WHO regional offices, as workers associated with these bodies, with different types of training, specialisation and institutional affiliation, frequently came up with varying ideas about how best to achieve different goals.^6^

To add to the complexity of what was really the first stage of policy implementation, departments within the different WHO offices would also often set up—on a collaborative basis or otherwise—specialist research groups to provide blueprints for action. These suggestions, which were often published as so-called technical reports, did not automatically become ordained as WHO blanket policy; instead, organisational representatives in the field were often directed by WHO office managers to give greater attention to some proposals than others, as a variety of political and economic considerations had to be made part of the larger calculations of designing and implementing policy. A further layer of operational complexity was added by the experiences of field personnel, who had to work in a variety of regional, national and local contexts. Indeed, as these officials—of different nationalities, races, gender and educational profile—adapted to a variety of political, economic, social and medical situations, they were forced to reinterpret centrally dictated policies in numerous ways. In doing this, it is striking that WHO field officials were continually forced, sometimes to their displeasure, to draw upon local sources of information and help. This assistance was generally sought from among local political structures and sections of the social groups at whom different public health policies were being targeted. This local knowledge and the resultant activities were, of course, not always in concert, as varying interests competed for recognition and precedence, adding several layers of operational complexity to the unfolding of public health and medical campaigns.^4^

It is worth noting here that all the WHO regional offices, their departments and the country representatives within them were important actors in the formulation and implementation of policy in the field. This has been ignored in most academic studies, which tend to focus on either the voices of a handful of people based in Geneva or the Health Assembly’s resolutions published by the WHO HQ after several rounds of careful editing. This also perhaps explains why the significant voices of national- and local-level staff, usually employed by different WHO offices on short-term contracts of varying lengths, is almost entirely lost in historical writings dealing with different health programmes.^8^ This is a serious lacuna, as the opinions and actions of such staff, who were usually in touch with local politicians and bureaucrats, acting as crucial links between them and a range of international WHO workers on a day-to-day basis, are a crucial element in projects sponsored, managed or encouraged by both the WHO HQ and regional offices. Getting access to these significant voices is difficult, requiring concerted archive research and a willingness to chase down personal papers and talk to WHO workers of all grades (sometimes in languages other than English). However, such difficulties should not be used by historians as a justification for the preparation of blinkered studies denying agency to all but a handful of senior WHO administrators.

## SOUTH ASIAN NATIONAL SMALLPOX ERADICATION PROGRAMMES

The WHO’s World Health Assembly (WHA) started considering the prospect of eradicating smallpox worldwide in early 1950—discussions on the topic were held within the WHA that year, and in 1953, 1954 and 1958. Indeed, Dr Brock Chisholm, the WHO’s first Director General, proposed global smallpox eradication in 1953, even if these discussions did not progress particularly far. Noticeable progress on the issue was witnessed at the 11th WHA, which was held in Minneapolis, USA, in 1958, where Professor Viktor Zdhanov, the USSR Deputy Minister of Health, argued that the eradication of the variola virus was theoretically possible and important to the world as a whole, including countries that had managed to expunge the disease within their territories. His views—and the proposal put forward by him in the shape of what is often referred to as the “Zdhanov resolution”—received broad-based support at the gathering, leading the WHO’s Executive Board to meet immediately after the WHA and announce preparations for a future smallpox eradication drive. In Geneva, this took the shape of the acceptance of donations of freeze-dried smallpox vaccine from the USSR and glycerolated vaccine from Cuba, which was used to create an “account” that would distribute stocks to countries where eradication campaigns were initiated; the decision also resulted in discussions with officials based in the WHO regional offices and national governments in charge of smallpox endemic territories.^2^

The relatively small number of WHO officials who started discussing the prospects of global eradication of smallpox in the early 1960s very much hoped that it would be a top–down campaign, wherein the WHO HQ in Geneva—and, particularly, some departments within it—would be able to set a general campaign agenda. Recommendations were, for instance, volunteered in relation to how immunisation might be carried out, what sort of vaccines to use and how to assess the achievement of eradication. However, their experience quickly revealed the pitfalls of believing that they could automatically assume such intellectual and technical leadership. Representatives within the WHO regional offices raised numerous queries about proposals sent in from Geneva, and highlighted their own firmly held belief that all central directives would require tailoring to fit local conditions. These features of “locality” were presented as being challenging and inconstant, which, in turn, it was argued, meant that programme implementation would require frequent re-jigging, as political arrangements with different national governments were set up, reconfigured or abandoned. Significantly, similar trends remained visible after 1967, when the WHO’s Health Assembly formally signed up, with great fanfare, to the goal of global smallpox eradication.^4^

There were disagreements, too, at other administrative levels about how a global campaign to eradicate variola might be organised and run. Plans that were presented as a good idea by one group of WHO workers at one regional office were almost routinely challenged within their organisation and outside. Criticism from within other regional offices was often quite strident, as officials based therein made it a point to underline the need to develop locally specific plans. And as the scope of what was defined as constituting the “locality” expanded from government structures located within specific national capitals to the political and social constituencies of the districts, subdivisions and villages within whose administrative confines immunisation policies were actually going to unfold, the disagreements within the complexity of WHO structures became even more marked.^9^

The South Asian subcontinent, which was the focus of the global eradication programme in the late 1960s and the 1970s owing to the high incidence of variola in the region, was a good case in point. WHO officials in touch with representatives of the Indian, Pakistani, Nepali, Sri Lankan, Bhutanese, Burmese and, later, Bangladeshi national governments—and, therefore, keenly aware of the many expectations and tensions within those multifaceted formations—refused to blindly accept orders relating to the blanket implementation of specific immunisation strategies and vaccine usage patterns coming in from Geneva. Strikingly, suggestions from the HQ were frequently queried and discussions were held within the regional offices about how the dictates from Geneva might be restructured to best meet a host of local needs. These trends were very noticeable within the Eastern Mediterranean Regional Office (EMRO), which dealt with Pakistan, and the South East Asian Regional Office (SEARO), which was charged with the task of working with the other subcontinental governments (including Bangladesh after 1971). An assessment of all such discussions, which is best done through a study of unpublished telegrams, letters and reports available in the various WHO archives, reveals that officials located within different levels and departments of the regional offices continued to hold disparate views right until global smallpox eradication was formally certified.^4^ ^5^

As is to be expected, the prevalence of numerous ideas about how work ought to be carried out within SEARO and EMRO influenced the many ways in which eradication policies were implemented. Like the WHO HQ in Geneva, the regional offices were not monolithic bodies. Some officials were more enthusiastic than others about the goal of variola eradication, and divergences in policy implementation were further encouraged by the fact that Regional Directors remained keen to advertise their autonomy by seeking to reconfigure guidelines received from the HQ, usually on the basis of their own understanding of local requirements. Such variation in bureaucratic support within the WHO was frequently identified in internal, unpublished documents as a significant impediment to the smooth running of the overall programme. This helps explain why SEARO structures were reorganised in the 1970s, clearly in an effort to ensure smoother and direct interactions between the Smallpox Eradication Unit headed by Donald Henderson in Geneva and the field officers in the region. Notably, this took the form of setting up a unit in New Delhi, within the SEARO establishment, which was put in Nicole Grasset’s charge; this body was made directly answerable to Henderson and his team and also given access to special funds donated by a variety of funding agencies (the Swedish International Development Agency was a major contributor towards the costs of the so-called intensified phase of activity in India and Bangladesh in the 1970s). The aim, it appears, was to counteract the then SEARO Regional Director’s opposition to the way the smallpox programme was being run in South Asia, and develop a relatively independent taskforce drawn from a variety of WHO-affiliated workers, both international and South Asian.^4^

This reorganisation of personnel helped in other ways as well. For example, it allowed for the inflow of a miscellany of ideas from the field about how best to adapt to a variety of local conditions (this information was often forthcoming from South Asian field officials of different ranks, who were involved in great numbers on contracts of varying lengths). Placed in the hands of Geneva- and New Delhi-based managers who were willing to avoid the strict top-down imposition of centrally dictated policies, to negotiate with the target population and, not least, to adapt work to assuage local concerns and innovate in relation to the running of the so-called search and containment strategies that were central to the campaigns of the 1970s, such input was invaluable. Indeed, it allowed teams of international and local workers, who were generally mobilised in groups containing personnel of different nationalities (the Indian government insisted on such an arrangement before allowing foreign epidemiologists to work in the country), to respond quickly to a diversity of local crises and social, political and economic needs.^10^ That the personnel were spared the need to get endless bureaucratic clearances for finances controlled by the Regional Director and national governments helped enormously, as it saved valuable time and allowed for greater flexibility.^4^ ^9^

This is not to say that opposition, from within WHO agencies and complex national political frameworks, disappeared completely over time. Indeed, pockets of often intense hostility remained in a situation where the Regional Directors retained powerful political alliances within and across national borders; this was compounded by the significant power held by critics within South Asian national, provincial and district governments and their various departments, and the doubts about the efficacy of vaccination harboured by some sections of society.^11^ Strikingly, not all public health and medical officials were supportive of smallpox eradication, as many considered the goal an impossible one and, therefore, a misguided waste of scarce resources. Administrative bottlenecks frequently resulted, as plans suggested by the WHO’s smallpox eradication units in Geneva and New Delhi were questioned and, sometimes, blocked within different levels of South Asian administration. These trends threw up vital challenges in a situation where WHO officials had varying levels of access to different national territories; problems that, it has to be noted, could be overcome only through sustained negotiations with politicians and bureaucrats of all ranks (including members of the political opposition), and members of the target population. As mentioned earlier, international workers could not just fly into the national capitals and then disperse as they wished. In all cases, they required clearance from a country’s federal authority for entry and work, with additional paperwork required for visits to politically sensitive enclaves (India’s North Eastern Frontier Area, as it was then designated, was a case in point, as was the highly disturbed Indo-Bangladeshi border in the 1970s).

The result, therefore, was a complex patchwork of distinct plans and patterns of work in a multiplicity of urban and rural areas. These coexisted uneasily, and sometimes openly came into conflict owing to the influence of a variety of administrative, economic and social factors; situations that required careful resolution through sensitive diplomatic negotiations carried out by WHO workers in association with their allies in national and local government. Force was sometimes used to counter opposition to vaccinations associated with search and containment regimes, but these were exceptions rather than the norm. Once again, these initiatives could not be carried out by WHO personnel in isolation, as the danger of a violent social and political backlash was acute—unpublished WHO and government correspondence regarding campaigns of forcible immunisation suggests careful planning and synchronisation of efforts between organisational employees, South Asian politicians of all ranks and hues and, not least, national and local military, paramilitary and police forces (links that were almost universally downplayed, by all involved, once smallpox eradication had been achieved). It was a combination of all these initiatives that allowed for the eradication of smallpox in South Asia, which was crucial to the ultimate removal of the disease globally.^4^ ^5^ ^12^

## CONCLUDING COMMENTS

The global eradication of smallpox is, by any measure, an enormous achievement. To recognise that this goal was reached in the face of tremendous difficulties, often emanating from within the organisations involved in the planning and implementation of policies, does not detract from that accomplishment. However, it does serve as a reminder that scholars should avoid being swept away by the heroic narratives that tend to predominate in official histories prepared after the certification of eradication. Historians and other chroniclers need to be equally careful about being over-reliant on reports published during the programme’s earlier stages, as these tend to offer only the views of a few people, who hoped, usually in vain, that their recommendations would be implemented as policy in the field. Ground realities, as this article attempts to show, were always significantly more intricate. And this complexity can only really be revealed by a careful analysis of unpublished papers dealing with the day-to-day discussions about policy, which are useful precisely because they reveal the views and actions of the thousands of field managers and personnel who contributed to smallpox eradication; their ability to study and adapt to a plethora of local conditions was crucial to the ultimate result and, therefore, merits recognition.

Assessing the intellectual, political and social agendas of a handful of senior WHO officials is fine as long as we do not end up assuming that everyone else associated with it was devoid of both intellect and the ability to make a difference in the design and implementation of policy. The views of WHO Directors General, their advisors and overall heads of disease control programmes are undoubtedly important. Yet, it is important to remember that their views were neither static nor able to dictate the day-to-day running of a highly complex organisation. At the same time, it would be foolhardy for the historian seeking to study the complex interplay between global, regional, national and local forces to ignore the complicated political networks that different constituents of the WHO had to contend with on a daily basis, often through the offices of staff employed locally on a variety of short-term contracts.^13^

The attempt here is to emphasise the difference between theory and practice; the need to distinguish between the official rhetoric from the WHO HQ and regional offices and the nature of work actually carried out in a variety of field situations is of paramount importance. This would allow the preparation of more rounded histories of health campaigns run on a global scale, which were—and continue to be—reliant on the assistance provided by numerous local political and social actors. And unlike some relatively thinly researched and jargon-filled analyses of the thoughts and actions of a few senior organisational personnel,^3^ ^8^ a thorough assessment of the intricacies of global health organisations and their links to national and local governments can actually provide useful insights into the management of current health programmes. Apart from anything else, the careful examination of policy implementation would suggest that the acute differences between vertical and horizontal health programmes, which analysts dependent on published policy assertions regularly allude to,^14^ ^15^ are far less marked than assumed. Indeed, local infrastructural exigencies and field experiences often forced developments that blurred the lines between preventive and curative medicine; an important point to remember when the WHO HQ’s renewed emphasis on the worldwide regeneration of the structures of primary healthcare is stoking interesting discussions, within and outside the organisation, about its ability to bring about meaningful changes in developing, less developed and developed countries.

**What this study adds**

A new historical perspective on the World Health Organization and its multifaceted involvement in the global smallpox eradication programme, which is one of the greatest medical achievements of the twentieth century.

**Policy implications**

This article suggests the adoption of historical methodology that can, the author feels, help prepare nuanced studies that can provide insights into ways of developing flexible and multifaceted international and global health campaigns.
